# The downside of aggressive volume administration in critically ill patients—“aggressive” may lead to “excessive”

**DOI:** 10.1186/s40560-019-0360-x

**Published:** 2019-02-02

**Authors:** Kenichiro Morisawa, Shigeki Fujitani, Yasuhiko Taira

**Affiliations:** Department of Emergency and Critical Care Medicine, St. Marianna University School of Medicine, 2-16-1, Sugao, Miyamae-ku, Kawasaki-shi, Kanagawa Japan

**Keywords:** Aggressive volume administration, Volume restriction, Volume resuscitation, Critical care, Intensive care unit

## Abstract

Management of fluid therapy in an intensive care unit (ICU) tends to be volume restriction after initial fluid resuscitation, since it has been the consensus that volume overload is associated with complications and poor clinical outcomes. Aggressive volume administration without cautious monitoring should be avoided in the ICU, because it could lead to excessive volume administration. However, there are limited consensus on determining the completion of resuscitation phase, in other words, when to stop aggressive infusion and initiate infusion restriction.

## Background

Aggressive volume administration in an intensive care unit (ICU) setting is necessary to maintain organ perfusion in critically ill patients. However, it has been the consensus that volume overload is associated with complications and poor clinical outcomes. In this article, we evaluate the risks associated with aggressive volume administration which may cause volume overload.

## Main text

### Aggressive volume administration with EGDT

In the management of sepsis treatment, the Surviving Sepsis Campaign Guideline (SSCG) recommends fluid resuscitation protocol with central venous pressure (CVP) monitoring, early goal-directed therapy (EGDT). One of the goals of EGDT is to prevent microcirculatory failure, tissue hypoxia, and organ dysfunction caused by aggressive volume administration [1]. However, a recent trio of trials comparing the effect of sepsis management between EGDT and commonly practiced “usual care” with less fluid infusion than EGDT, concluded that EGDT did not significantly reduce mortality compared to usual care [2–4]. It is noteworthy that the amount of fluid given in the first 6 h was less in the usual care protocol without CVP monitoring than in the EGDT including CVP monitoring (2279 vs 2805 ml) [3]. Furthermore, almost 50% of patients in the EGDT group had achieved CVPs greater than the stated goal (> 8 to 12 mmHg) [3]. One of the reasons that EGDT failed to show usefulness compared with usual care could be the fact that CVP might not adequately reflect hemodynamic changes and might lead to excessive fluid infusion following aggressive volume administration [5–7].

### Effectiveness of restrictive fluid management

Volume overload leads to poor outcomes in septic patients [8]. The study with adult septic shock patients reported that fluid-restricting protocol successfully reduced fluid volume at day 5 and during ICU stay, without adverse events, such as ischemic events, acute kidney injury events, or death within 90 days of admission [9]. A systematic review, which included 19,902 critically ill patients compared restrictive strategy attempting to obtain a neutral or negative cumulative fluid balance to unrestricting strategy not attempting to obtain negative fluid balance after the third day of ICU. This review showed that restrictive fluid management strategy resulted in less positive cumulative fluid balance of 5.6 L compared to controls after 1 week of ICU stay and was associated with lower mortality rate compared to patients treated with unrestricted fluid management strategy (24.7% vs 33.2%) [10].

Furthermore, several studies also demonstrated the effectiveness of neutral or negative cumulative fluid balance in certain patient groups, excluding patients with sepsis.

#### Central nervous system

A randomized controlled study (RCT) which included 32 patients with subarachnoid hemorrhage reported that the hyperdynamic therapy group suffered more complications, including congestive heart failure combined with arrhythmia and pulmonary edema, and higher costs [11]. In a retrospective study of patients with subarachnoid hemorrhage, positive fluid balance was associated with increased odds of vasospasm and prolonged length of hospital stay [12].

#### Cardiovascular system

In a prospective cohort study with 1770 critically ill patients, a new-onset atrial fibrillation (AF) was seen in patients with significantly greater net-positive cumulative fluid balance, compared to those without AF. Any AF in the ICU was associated with increased mortality and prolonged duration of illness [13].

#### Respiratory system

A randomized study included 1000 patients with acute lung injury reported that volume restriction was found to be effective. Their cumulative fluid balance during the first 7 days was significantly reduced in the conservative strategy (volume restriction) group compared to the unrestricted volume infusion strategy group. Compared to the unrestricted strategy group, the conservative strategy group improved the oxygenation index, the lung injury score, the number of ventilator-free days, and the length of ICU stay, without increasing adverse events such as shock, or requirement of renal replacement therapy [14].

#### Renal system

In 618 adults with acute kidney injury with or without the requirement of dialysis, patients with fluid overload defined as more than a 10% increase in body weight relative to baseline, experienced significantly higher mortality rate within 60 days [15]. As Malbrain et al. reported, the pathophysiologic adverse effects of fluid overload could affect almost all end-organ functions, central nervous, respiratory, cardiovascular, hepatic, renal, gastro-intestinal, abdominal wall, and endocrine system [10].

### Validity of volume resuscitation

On the other hand, a hypovolemic state and reduced organ perfusion secondary to increased vascular permeability requires adequate fluid administration. This is a fundamental element in the management of critically ill patients, especially in the acute phase. Thus, the patients with restrictive protocol reported by Hjortrup et al. had received at least 30 ml/kg of crystalloid fluid before randomization [9]. The four phases of intravenous fluid management as a conceptual model have been previously discussed [16]. The recent recommended restrictive infusion is, after a sufficient infusion with the controlled aggressive volume administration in the acute phase, to restrict infusion in the stable phase (“stabilization phase” and “de-escalation phase”), so-called “usual care” in common practice.

### Difficulty to initiate restrictive strategy

The question remains on when to initiate infusion restriction. Theoretically, if volume restriction is initiated immediately upon completion of the resuscitation phase, unnecessary volume administration may be avoided. There are a few gold-standard methods to determine when resuscitation phase is completed. Meta-analysis including 11 studies, reported the efficacy and safety of conservative fluid strategies in adults and children in the post-resuscitation phase of critical illness [17]. In this meta-analysis, only one study defined the end point of volume resuscitation [18]. To avoid relying on each intensivist’s judgment based on experience, it is necessary to standardize and determine the end point of resuscitation infusion, as well as establish means to monitor early recognition of decreased organ perfusion due to infusion restriction.

## Conclusions

In conclusion, aggressive volume administration should be controlled to avoid volume overload-associated complications. Volume restriction should be initiated promptly in the stabilization phase to reduce the potential for volume overload. However, it remains uncertain how clinicians decide the end of volume resuscitation phase. It also remains controversial when clinicians should initiate infusion restriction strategy.

## Abbreviations

CVP: Central venous pressure
EGDT: Early goal-directed therapy
ICU: Intensive care unit
RCT: Randomized controlled study
SSCG: Surviving sepsis campaign guideline
